# Water discharge variations control fluvial stratigraphic architecture in the Middle Eocene Escanilla formation, Spain

**DOI:** 10.1038/s41598-023-33600-6

**Published:** 2023-04-26

**Authors:** Nikhil Sharma, Alexander C. Whittaker, Stephen E. Watkins, Luis Valero, Jean Vérité, Cai Puigdefabregas, Thierry Adatte, Miguel Garcés, François Guillocheau, Sébastien Castelltort

**Affiliations:** 1 Department of Earth Sciences, University of Geneva, 13 Rue des Maraîchers, 1205 Geneva, Switzerland; 2 Department of Earth Science and Engineering, Imperial College London, South Kensington, London, SW7 2AZ UK; 3 Paleomagnetic Laboratory CCiTUB-Geo3Bcn, Geosciences Barcelona–CSIC, C/Lluis Solé I Sabarís S/N, 08028 Barcelona, Spain; 4 LPG – Le Mans, UFR Sciences Et Techniques, Université du Maine, 72089 Le Mans Cedex 9, France; 5 Department of Earth and Ocean Dynamics, Faculty of Earth Sciences, Universitat de Barcelona, C/ Martí I Franquès, S/N, 08028 Barcelona, Spain; 6 Institute of Earth Sciences (ISTE), University of Lausanne, Bâtiment Géopolis, 1015 Lausanne, Switzerland; 7 UB-Geomodels Research Institute, Universitat de Barcelona, 08028 Barcelona, Spain; 8 Géosciences Rennes, Université de Rennes 1, Campus de Beaulieu, 35042 Rennes Cedex, France

**Keywords:** Climate sciences, Hydrology

## Abstract

Ancient fluvial deposits typically display repetitive changes in their depositional architecture such as alternating intervals of coarse-grained highly amalgamated (HA), laterally-stacked, channel bodies, and finer-grained less amalgamated (LA), vertically-stacked, channels encased in floodplain deposits. Such patterns are usually ascribed to slower, respectively higher, rates of base level rise (accommodation). However, “upstream” factors such as water discharge and sediment flux also play a potential role in determining stratigraphic architecture, yet this possibility has never been tested despite the recent advances in the field of palaeohydraulic reconstructions from fluvial accumulations. Here, we chronicle riverbed gradient evolution within three Middle Eocene (~ 40 Ma) fluvial HA-LA sequences in the Escanilla Formation in the south-Pyrenean foreland basin. This work documents, for the first time in a fossil fluvial system, how the ancient riverbed systematically evolved from lower slopes in coarser-grained HA intervals, and higher slopes in finer-grained LA intervals, suggesting that bed slope changes were determined primarily by climate-controlled water discharge variations rather than base level changes as often hypothesized. This highlights the important connection between climate and landscape evolution and has fundamental implications for our ability to reconstruct ancient hydroclimates from the interpretation of fluvial sedimentary sequences.

## Introduction

**Figure 1:**
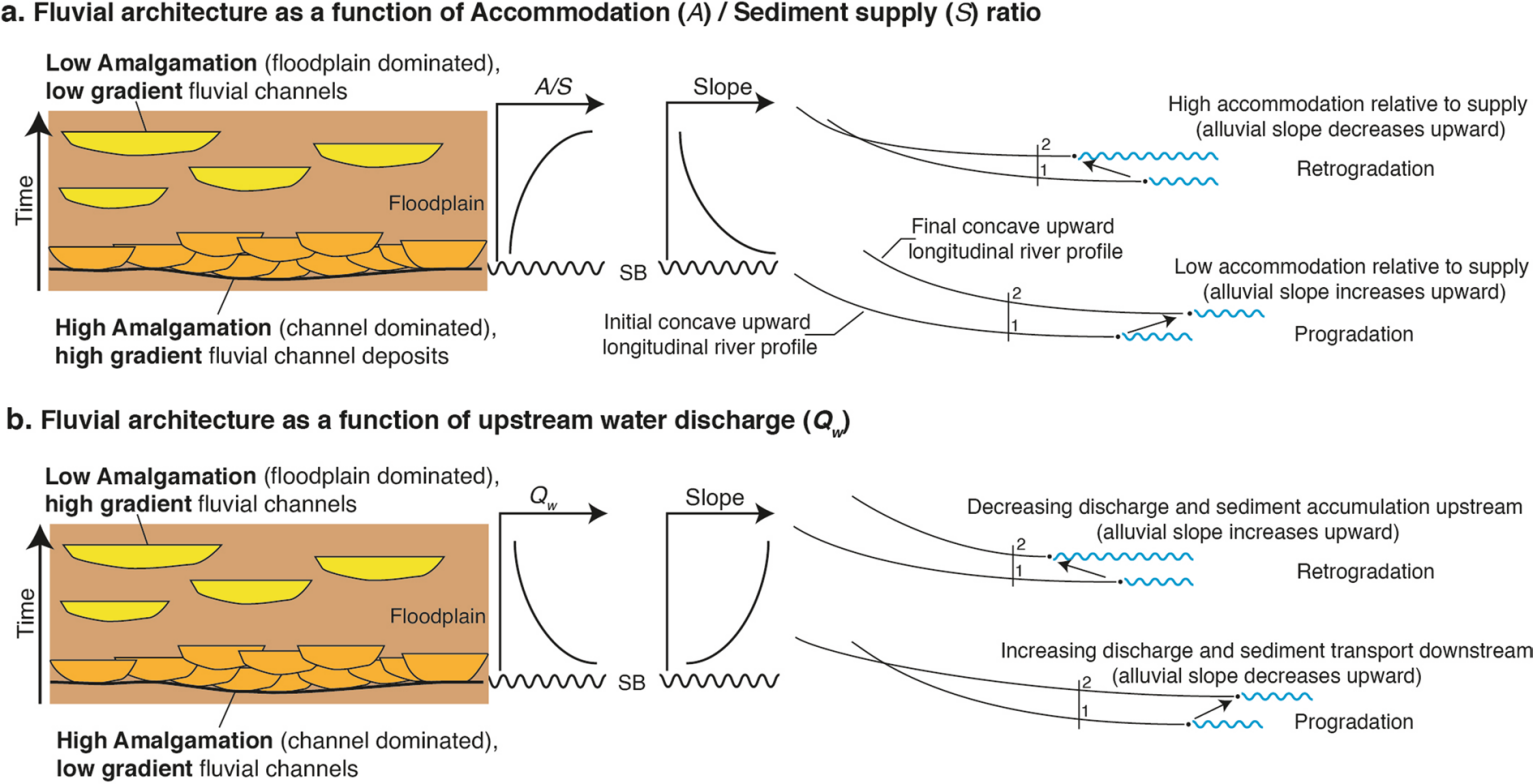
Conceptual figure explaining fluvial architecture as a function of the Accommodation (*A*) to Sediment supply (*S*) ratio and water discharge (*Q*_*w*_). (**a**) Low *A/S* results in the deposition of high gradient fluvial channels with a high degree of amalgamation and an overall progradation of the system. Under high *A/S*, low gradient fluvial channels with a low degree of amalgamation are deposited with an overall retrogradation of the system. Figure based on the ideas by e.g.,^4,10^ (**b**) As *Q*_*w*_ increases, low gradient channels with a high degree of amalgamation are deposited with an overall progradation of the system while as *Q*_*w*_ decreases, high gradient channels with a low degree of amalgamation are deposited with an overall retrogradation of the system. In this scenario, base level rise is constant and changes in fluvial architecture are solely driven by changes in *Q*_*w*_.

An assemblage of fluvial deposits such as vertically stacked isolated channels and laterally extensive amalgamated channels reflects the complex interplay of various factors such as climate, tectonics, and base level fluctuations^1–3^. In theory, both “downstream” i.e., base level changes which can be relative sea-level representing the joint effect of eustasy and tectonics (local/regional subsidence rates), or a stratigraphic reference level above which sub-aerial erosion prevails^4^, and “upstream” factors i.e., sediment flux (*syn. Sediment transport*), sediment size, and water discharge (*syn.* streamflow, channel runoff), have been recognised for their ability to determine patterns of channel-floodplain sequential arrangements^5–10^. For instance, in the downstream sectors of a fluvial system, the historical approach involving stratigraphic base level^1,7,10,11^ considers the interplay of two rates—the rate of change of accommodation space, hereafter referred to as *A*, i.e., the space available for sedimentation, and the rate of sediment supply, hereafter referred to as *S*. In this view, the resulting balance in the form of *A/S* is the predominant factor controlling sediment depositional architecture [e.g.,^12^]. In practice, changes in the stacking pattern of alluvial/fluvial conglomerates and sandstone are often interpreted as changes in *A/S* (Fig. 1a), with laterally stacked strata interpreted as being deposited under low *A/S* (High Amalgamation HA intervals) while vertically stacked strata are interpreted as being deposited under high *A/S* (Low Amalgamation LA intervals) [e.g.,^6,10^]. Similarly, Wright & Marriott^7^ in their sequence stratigraphic model proposed the deposition of multistorey sand bodies under low *A*, while vertically stacked isolated channels encased into thick floodplain deposits would form during periods of increasing *A*. Although the role of *S* in sequence stratigraphy is widely acknowledged^13,14^, sequence stratigraphic interpretations are often based on the primacy of base level controlled *A*, due in part to independent knowledge of sea-level variations (e.g.,^15^, and to the inherent difficulties of reconstructing *S*^16^.

While many historical approaches consider downstream factors fundamental in controlling the long profile and sedimentary record of alluvial rivers, several studies^10,17–19^ have demonstrated that upstream factors have an influence over much of the river profile. For alluvial rivers, where base level variations are not the only dominant control on creation of *A*, one must consider the resulting equilibrium profile^1^. For instance, the early work of fluvial geomorphologists such as Lane^20^ and Leopold & Bull^21^ has indicated that the equilibrium river profile and thus channel slope is a function of upstream boundary conditions of sediment flux, sediment size, and water discharge. Experimental studies such as numerical modelling and sedimentary forward modelling studies too have recognised the role of upstream factors in modifying the river profile^19,22–24^. Through a series of numerical experiments, Simpson & Castelltort^22^ highlighted the evolution in slope of a river profile under sinusoidal water flux variations such that river profile gradient increases as water flux decreases and the gradient decreases as water discharge increases. In terms of stratigraphic architecture, this could result in low gradient high amalgamation (HA) intervals being the dominant landform deposited under high water discharge conditions, while high gradient low amalgamation (LA) intervals would be dominant under low water discharge context (Fig. 1b). Field-based studies in the past [e.g.,^25,26^] have documented water discharge variations under orbital forcing parameters as the primary control on varying thicknesses of fining-upward sequences in Devonian-aged fluvial section from East Greenland, while more recent studies such as Noorbergen et al.^27^ have pointed at enhanced discharge and sediment supply during seasonal conditions under increasing eccentricity in affecting fluvial architecture and sedimentation patterns in continental settings^27^.

Channel slope evolution in this framework depends on whether the sequences are dominantly controlled by accommodation *A* or sediment supply *S*. In *A*-controlled sequences, the LA stacking pattern presents lower alluvial slopes, while the HA stacking pattern displays higher channel slopes (Fig. 1a). *S*-controlled sequences on the other hand have LA intervals with higher slope while HA intervals have lower slope (Fig. 1b). Channel slope evolution may thus be seen as a potential diagnostic tool to distinguish between upstream palaeo-environmental drivers on the stratigraphic record relative to a base level control. An outstanding research challenge in this field, therefore, lies in deciphering the factors controlling these changes in observed fluvial architecture at a range of temporal and spatial scales. Field-based work such as Foreman et al.^28^ and Lyster et al.^29^ along with empirical studies based on flume experiments and modern river systems^30,31^ have demonstrated the ability to meaningfully quantify palaeohydrological parameters, including channel gradients, from the rock record. These include the development of tools to estimate palaeoslope^31,32^, and other palaeohydrological parameters such as flow velocity, water discharge and sediment flux.

Yet, to our knowledge, the evolution of river slope across fluvial sequences in relation to documented cyclical changes in stratigraphic architecture has never been explored. In this work, we address this problem using the well-documented Middle Eocene Escanilla Formation in Spain^33–35^ to explore the drivers of fluvial stratigraphic cyclicity and the environmental factors they record. We estimate channel slope evolution, along with documenting channel-belt widths and style (sheet or ribbon), flow velocity, water discharge and sediment flux, across several HA-LA stratigraphic cycles. Palaeohydraulic estimates show a systematic increase (*resp.* decrease) in discharge and sedimentary fluxes during HA (*resp*. LA) intervals, thereby pointing towards an upstream-driven climate control on water discharge in channels and stratigraphic architecture, that we discuss in relation to Earth’s orbital cycles.

## Geological setting and stratigraphy

### The Escanilla sediment routing system

**Figure 2:**
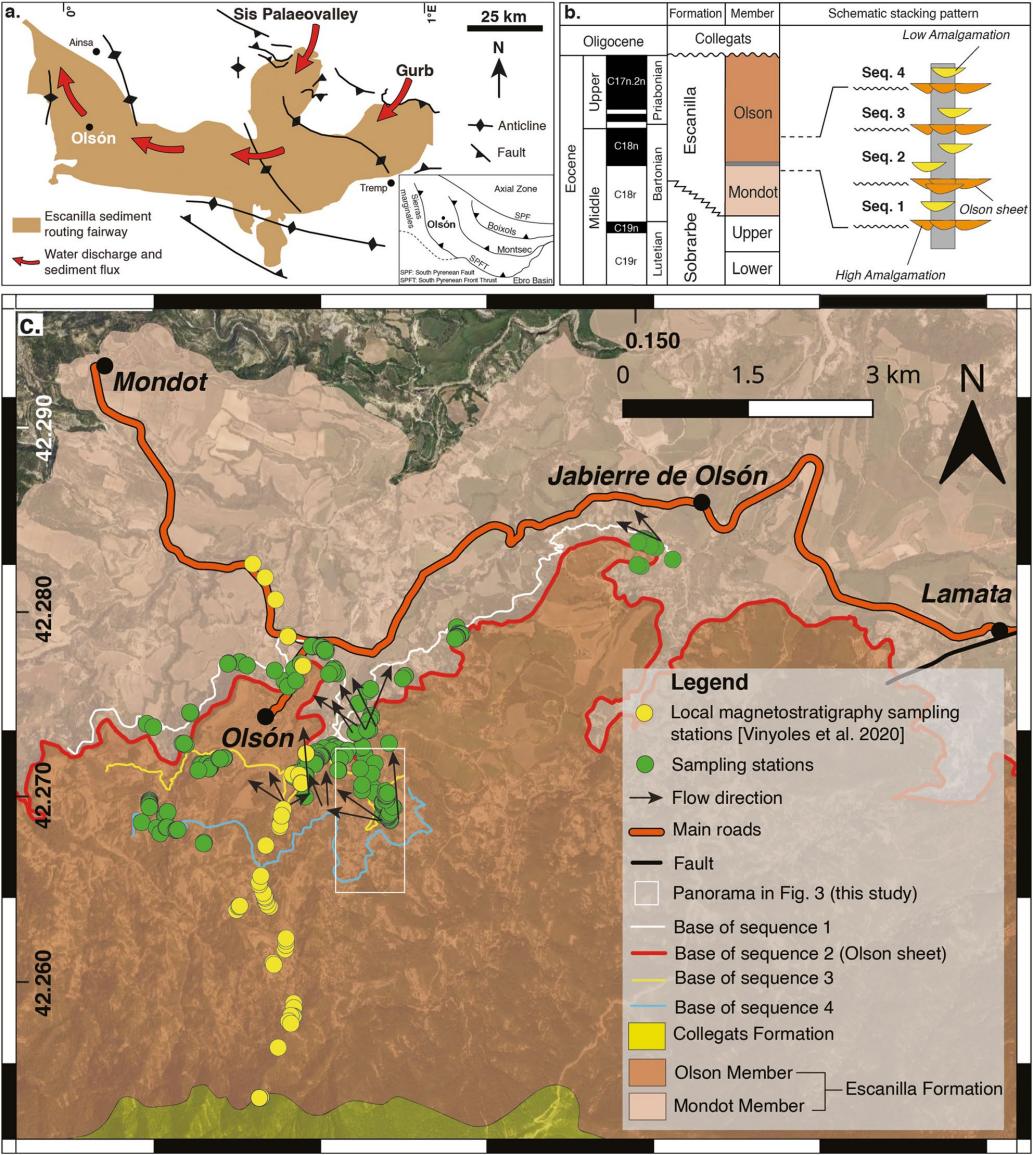
Geological setting of the Escanilla sediment routing system and the Escanilla Formation at Olson. (**a**) Geological setting of the South-Pyrenean Foreland basin containing the Escanilla sediment routing fairway. Red arrows mark the sediment transport direction of the Escanilla system from the source regions of Sis and Gurb (**b**) Lithostratigraphic framework^40,41^ of the Escanilla Formation at Olson consists of two main Members – the Mondot and Olson Members with the ‘Olson sheet’ lying at the transition between the two Members. Also displayed is the magnetostratigraphic correlation^42^ and the schematic stacking pattern along the studied section. It is noteworthy that the thickest normal magnetozone represents Chron C18n, which includes C18n.1n + C18n.1r + C18.2n (the very short C18n.1r is missing) (**c**). Geological map of the southern Ainsa basin encompassing the Escanilla Formation around the village of Olson where the study area lies. Sampling stations are displayed along with flow directions with respect to the sampled sequences. The ‘Olson sheet’ is marked in red as a basin wide, laterally extensive amalgamated channel body lying in-between the Mondot and Olson Members of the Escanilla Formation. A Google Earth panel with locations of sampling stations has been provided in the supplementary material as Figure S1. This map was prepared using QGIS Desktop 3.22.8 (https://qgis.org/en/site/).

The Escanilla Formation corresponds to an ancient sediment routing system of late Lutetian to late Priabonian age, active between approximately 42 and 36 Ma in the south-Pyrenean foreland basin, Spain^36^. The Escanilla Formation was mainly sourced from the Pyrenean axial zone through large valleys filled with transverse alluvial fans, such as the fan system of the Sis palaeovalley^37^ and the Gurb escarpment^38^ further east (Fig. 2a). The maximum preserved thickness of the Escanilla Formation within the Ainsa basin is approximately 1000 m^34^, and is subdivided into two informal Members, the Mondot and Olson Members^39^ with a basin-wide extending conglomeratic channel-complex, often named the ‘Olson sheet’ at the transition between the two Members (Fig. 2b,c). Kjemperud et al.^33^ subdivided the Escanilla Formation into three units based on changes in alluvial geometry which are further subdivided into seven unconformity bound sequences. Similar alternating sequences have also been identified by^34,35^ as basin-wide, laterally extensive amalgamated channels underlying intervals of vertically-stacked isolated channels. We focus on exposures near Olson, where the gullied landscape and high-quality outcrop preservation allows a detailed documentation of stratigraphic architectural changes across three sequences, which correspond to sequences 2, 3 and 4 of Kjemperud et al. ^33^ and sequences 1, 2 and 3 of Labourdette & Jones^34^ and Labourdette. Based on the local magnetostratigraphy, the studied sequences represent several hundred thousands of years of deposition at approximately 40 Ma (Fig. 2b).

### Stratigraphic arrangement

**Figure 3:**
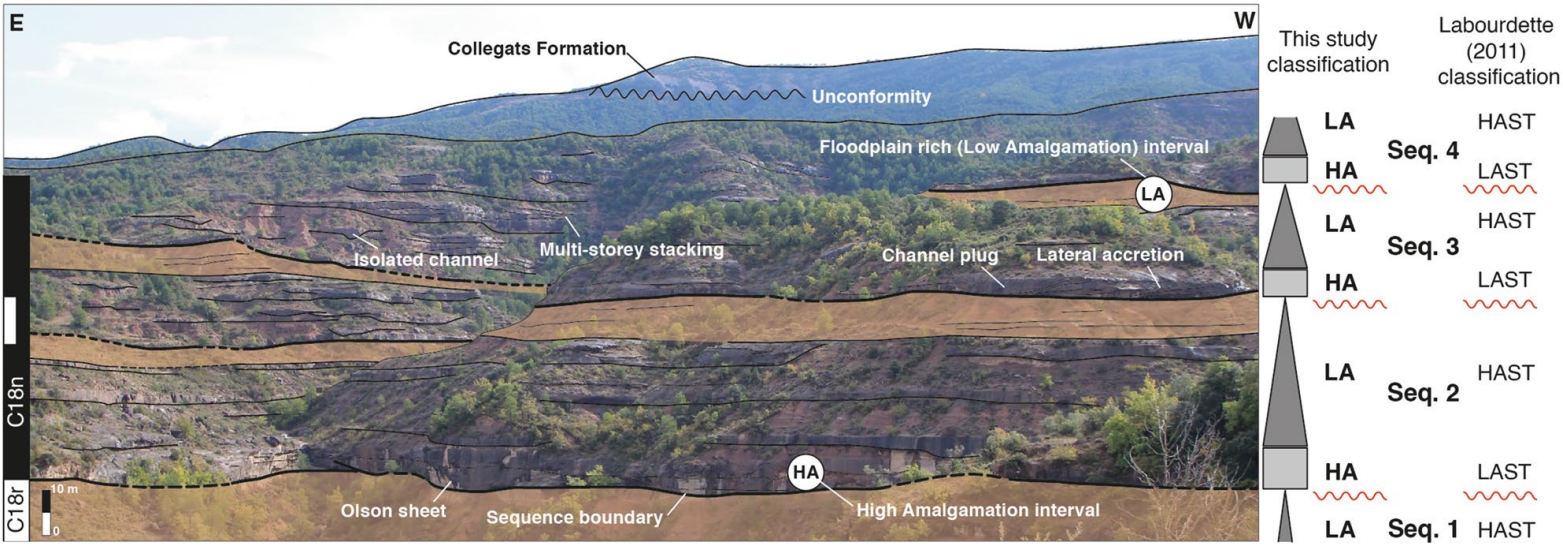
Field view of the studied sequences. This panorama depicts the studied intervals in sequences 2, 3 and 4 (sequence 1 lies below). At the base of the panorama lies a thick floodplain dominated interval terminating the LA interval of sequence 1, and above which lies the High Amalgamation (HA) interval corresponding to the ‘Olson sheet’. Above the HA interval lies the floodplain dominated Low Amalgamation (LA) interval. Several stratigraphic features such as channel plug, lateral accretion, isolated channel, and multistorey stacking pattern have been marked as well. To the top of the panorama lies the upper Eocene–Oligocene Collegats Formation separated from the underlying Escanilla Formation by an unconformity. Note that the HA-LA (High/Low Accommodation) packages used here correspond to the LAST-HAST packages (High/Low Accommodation Systems Tract of Labourdette^35^. An unannotated version of this panorama has been provided in the supplementary material as Figure S2.

**Figure 4:**
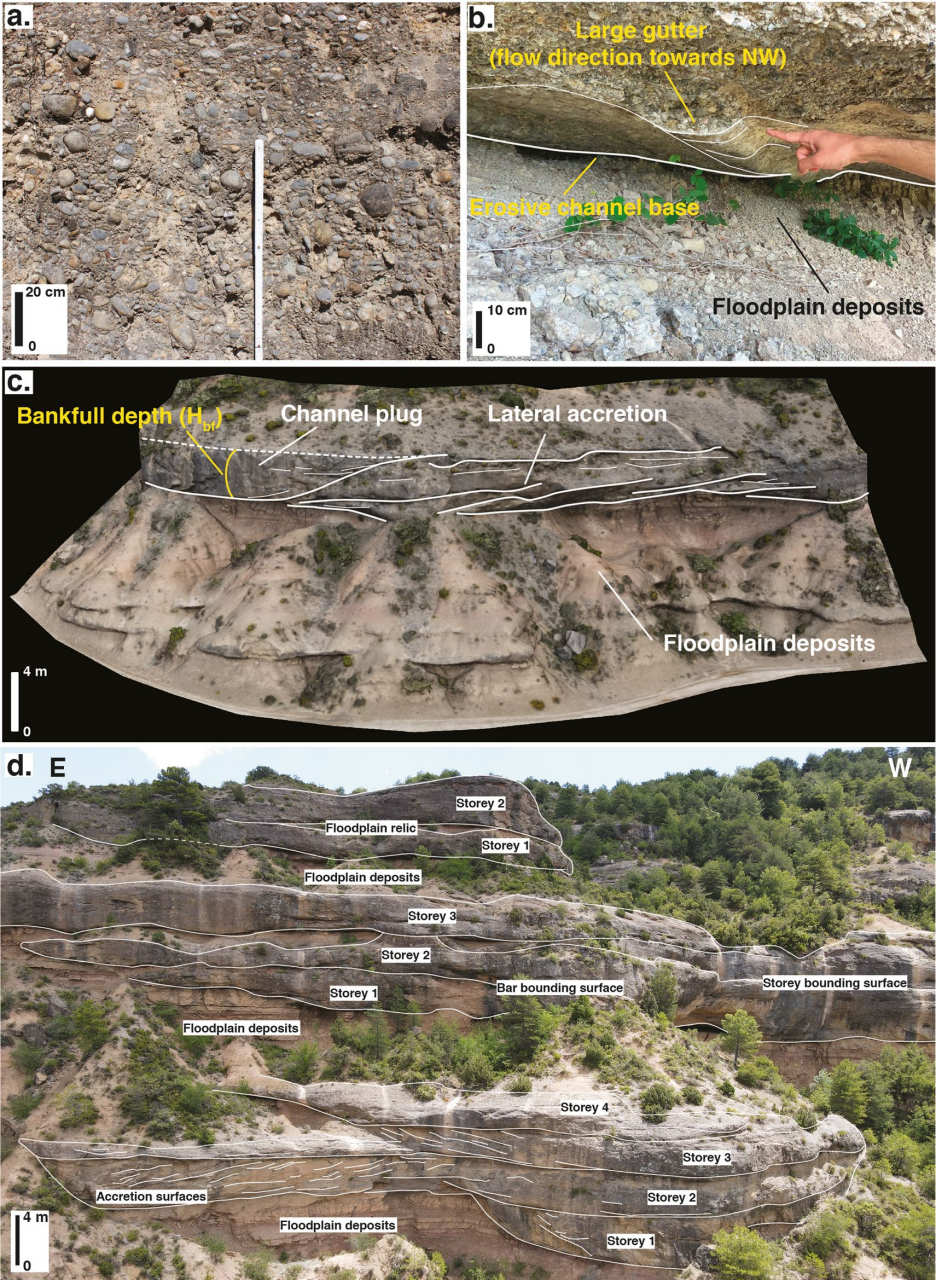
Outcrop photographs. (**a**) Channel basal gravel from which grain size estimates are obtained. (**b**) Example of a large gutter cast used to reconstruct flow direction in combination with pebble imbrications, base of a channel in the Olson Sheet (HA sequence 2) (**c**) 3D outcrop model showing a channel plug and lateral accretion deposits, which allow measuring bankfull height (H_bf_) (**d**) Multi-storey stacking pattern observed in the LA interval of sequence 4. An unannotated version of this photographic panel is provided as supplementary Figure S3.

This study focuses on 3 fining-upward sequences at Olson. Each fining-upward sequence consists of a High Amalgamation (HA) package which can be defined as a thick (5–12 m) and laterally extensive (~ 600–2000 m) channel-body complex (90% channel to 10% floodplain) typically consisting of multiple stories (1–4), and a Low Amalgamation (LA) interval which can be defined as a floodplain dominated (30% channel to 70% floodplain) interval with isolated channel bodies that are 2 – 4 m thick and approximately 100 – 500 m laterally extensive and typically consist of a single storey, although multiple stories can be found as well. On the field, the thickness of each such fining-upward sequence is around 35 – 45 m and a HA interval typically lies above a thick (~ 5 – 7 m) floodplain from the ‘LA’ interval of the underlying sequence (Fig. 3). Several different outcrop photographs can also be seen in Fig. 4^35^.

## Results

### Grain size, bankfull depth and palaeoslope evolution

**Figure 5:**
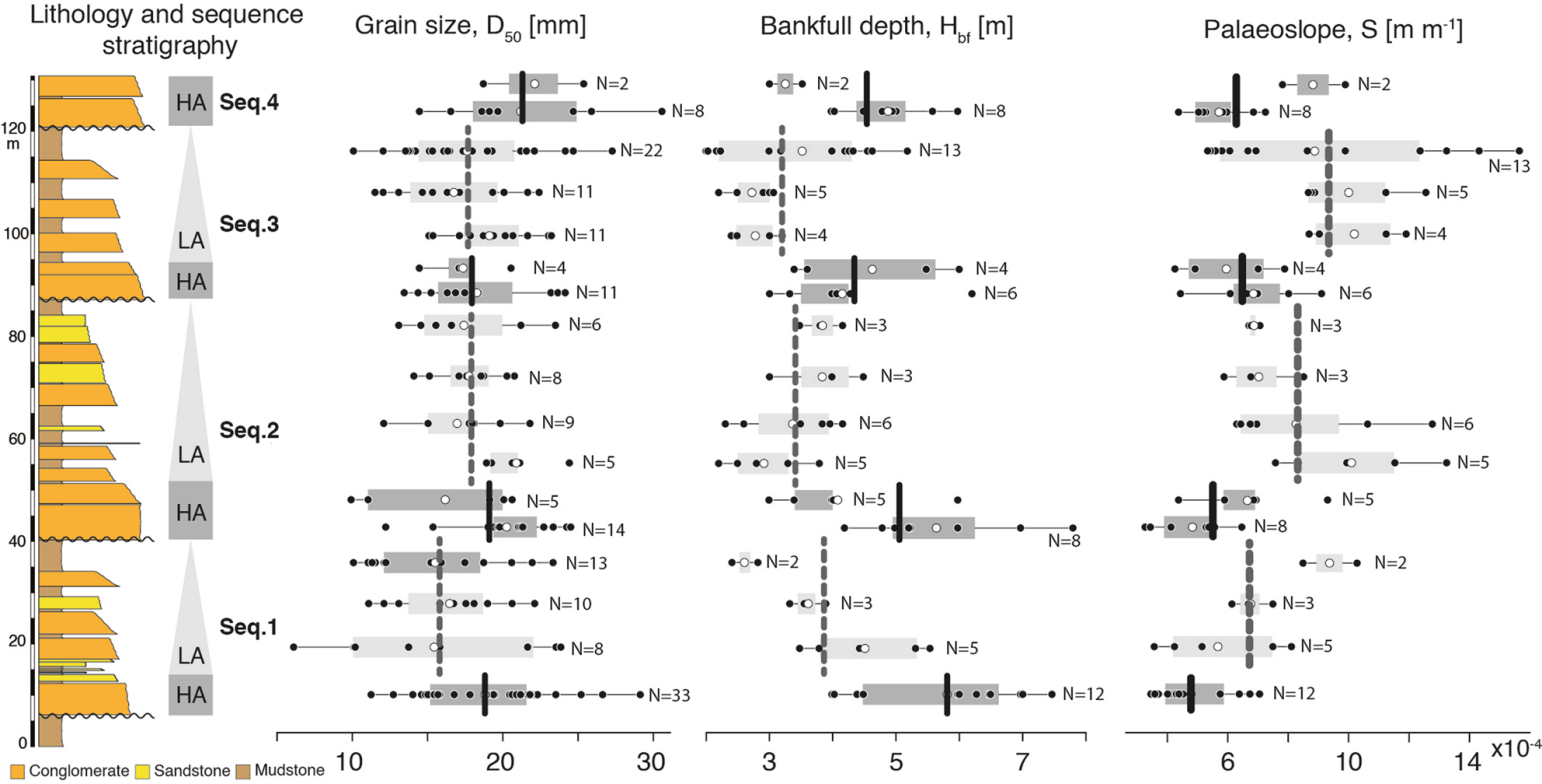
Grain-size, flow depth and palaeoslope evolution as a function of the degree of amalgamation. Stratigraphic log of the studied section depicting the three studied fining-upward HA-LA sequences. The calibration to the geomagnetic polarity timescale 2016^42,43^ is shown to the left. Median grain size, D_50_ [mm], channel bankfull depth, H_bf_ [m], and channel bed palaeoslope, S [m/m], are shown for each sampled fluvial storey. Black vertical bars denote the average value in HA intervals while grey bars denote the average value in LA intervals.

Coarse grain size fraction (> 4 mm) (Fig. 5) was measured at 180 stations distributed across the studied stratigraphic interval. Coarse grain size fraction in channel bodies has an average range of grain sizes from 6 ± 2 [mm] to 30 ± 2 [mm] over the studied stratigraphic interval (Fig. 5). At the scale of individual sequences, HA intervals have a grain size of 19 ± 1 [mm] (average value ± standard error, N = 77), while LA intervals have a grain size of 17 ± 1 [mm] (N = 103), indicating that channel body grain size is 2 mm (~ 10%) larger in channels with a high degree of amalgamation. This relatively small difference is nevertheless statistically significant at the 95% confidence level (*t* value = 2.98, *p* value = 0.003, power = 0.91, dof = 178). It is noteworthy that the bulk grain size of LA intervals would be significantly lower if the fine-grained sediments of the floodplain were taken into consideration (50 – 70% of LA intervals).

Bankfull depths based on preserved storey thickness (Fig. 5) reveal a trend of higher depths in the HA intervals and a substantial decrease in the LA intervals. HA intervals have a depth of 5.0 ± 0.4 [m] (average value ± standard error, N = 45), while LA intervals have a depth of 3.5 ± 0.3 [m] (N = 49), i.e., an increase of 40% of flow depth during deposition of the HA intervals. These field observations suggest that the palaeohydrology of channels comprising the HA and LA intervals is not the same. A *t* test on bankfull depth data rejects the null hypotheses that HA and LA intervals have the same average values at the 95% confidence level (*t* value = 6.83, p-value = 0.8e^−9^, power = 0.67, dof = 92).

Palaeoslope estimates, obtained using the equation proposed by Trampush et al.^31^ (Eq. 1), are consistently lower in the HA intervals, and markedly higher into the LA intervals (Fig. 5). Palaeoslope estimates based on averaged field grain size and channel depth data of 7 storeys within HA stratigraphic intervals (77 grain size sampling stations wherein 100 – 200 clasts were counted per station, and 45 channel depth estimates) have a palaeoslope of 5 × 10^−4^ ± 5 × 10^−5^ [m/m] (average value ± standard error, N = 7), equivalent to 0.03°, while 11 storeys within LA stratigraphic intervals (103 grain size sampling stations and 49 channel depth estimates) have a palaeoslope of 8 × 10^−4^ ± 6 × 10^−5^ [m/m] (N = 11), equivalent to 0.05°, and representing a 60% increase in slope in the LA interval. A *t* test on palaeoslope estimates (*t* value = −4.02, *p* value = 0.001, power = 0.16, dof = 15) rejects the null hypotheses that HA and LA intervals have the same average values at the 95% confidence level. It is important to remark that the data in Fig. 5 show considerable variability typical of a natural fluvial system, but which nevertheless highlights systematic differences between HA and LA stratigraphic patterns.

### Channel-belt width estimates and channel body geometry

**Figure 6:**
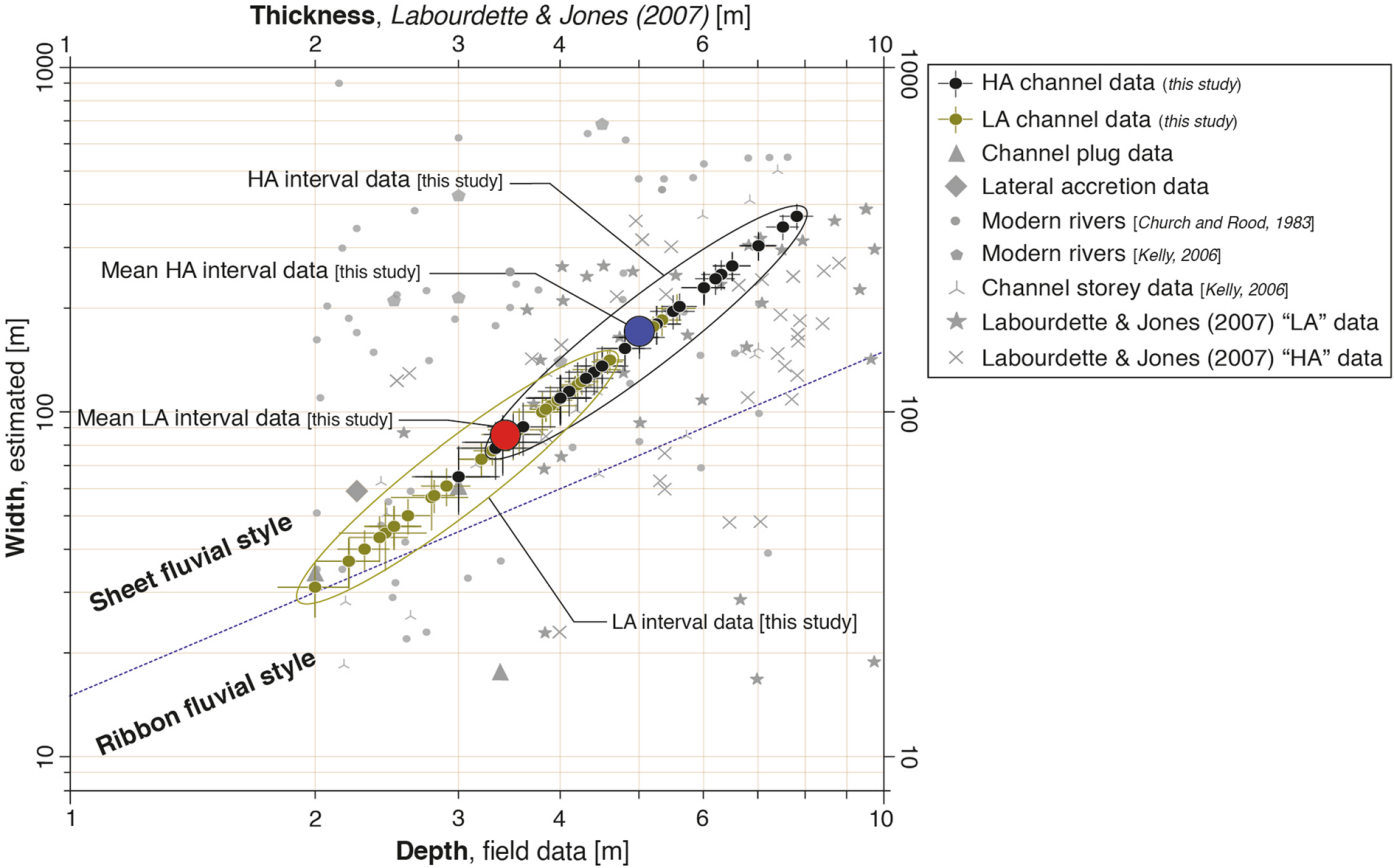
Comparison between modern river channel width and depth to estimates from this study. A cross plot with width and depth of modern rivers (data from^47^ and ^48^ having grain sizes between 5 – 45 mm and flow depths of 2 – 7.5 m which is the range of grain sizes and depths measured at Olson. Such a plot also allows the prediction of fluvial style during HA and LA intervals.

Channel-belt widths are necessary to estimating total discharge and sediment flux (e.g.,^29,44^. Despite excellent outcrop conditions, fully preserved channel cross-sections required to measure width in the field, are nevertheless difficult to assess due to their size and the 3D nature of the outcrops. To circumvent this limitation, we estimate channel-belt widths using the relationship proposed by Bridge & Mackey^45^ based on the model of Bridge & Leeder^46^, and compare them to width measurements of channel plug and lateral accretion deposits where preserved (Fig. 6). Results suggest that HA channel-belts are typically twice wider, 171 ± 22 [m] (average value ± standard error, N = 45) than LA channel belts, 86 ± 11 [m] (N = 49). These estimates are consistent with an empirical comparison to the width and depth of modern rivers having similar grain sizes (5 – 45 mm) and flow depths (2 – 7.5 m) (Fig. 6), which predicts active flow widths of HA channel belts near a central value of 180 m, in a range of 60 to 400 m, while the predicted flow width of LA channel belts is more likely near a central value of 90 m, in a range of 30 to 200 m. Both estimates are smaller than the “geobody” widths estimated by Labourdette & Jones^34^, and we hence consider them to represent adequate conservative values. Lastly, a cross plot of estimated channel belt depth and width in Olson primarily implies sheet-like channel geometry during HA intervals, while channel geometry during LA intervals, even though corresponds to sheet-like geometry, is more closer to the ribbon fluvial style with decreasing depth and width (Fig. 6).

### Palaeohydrology and sediment transport

**Figure 7:**
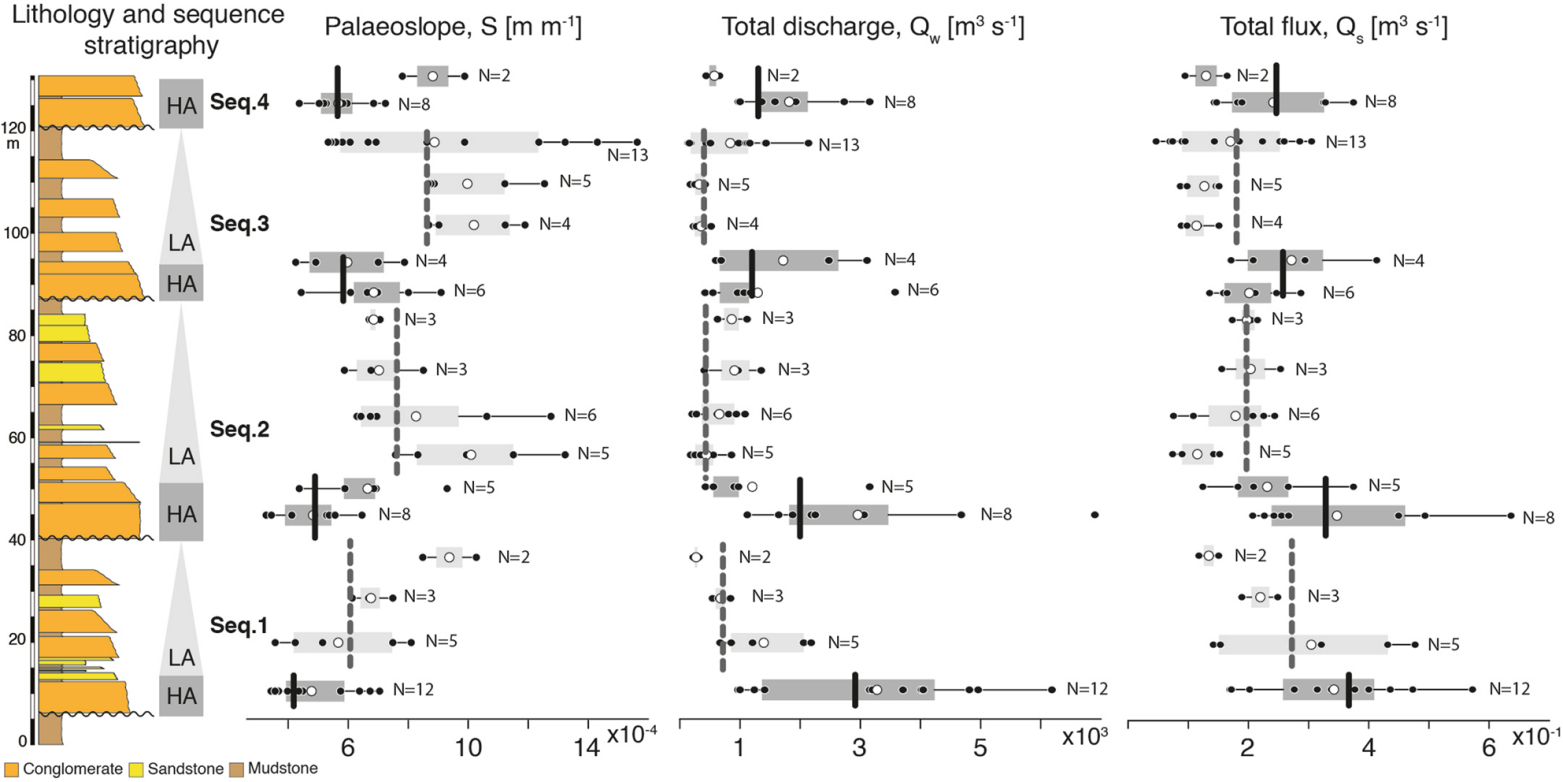
Palaeoslope, total water discharge and total bedload sediment flux estimates. Palaeoslope evolution along with estimates of total water discharge and total bedload sediment flux relative to each sampled storey within the HA and LA intervals of the studied sequences. Black vertical bars denote the average value in HA intervals while grey bars denote the average value in LA intervals. It is important to note the relationship and cyclical pattern, as shown by the overall average values in HA and LA intervals, between the three parameters such that river slope is lower when total discharge and total flux are higher (and conversely). Unit fluxes (water and sediment) have similar patterns of variations and are provided in supplementary figures S5 and S6.

Unit discharge (calculated by multiplying flow velocities by estimated depths) in HA intervals is found to be 11 ± 2 [m^2^ s^−1^] (mean value ± standard error, N = 45) and 7 ± 1 [m^2^ s^−1^] (N = 49) in LA intervals (supplementary material Figure S5). Multiplying unit discharge by channel-belt width estimates implies a total discharge rate of 2200 ± 550 [m^3^ s^−1^] (average value ± standard error, N = 45) in HA intervals, and a discharge rate of 700 ± 200 [m^3^ s^−1^] (N = 49) in the LA intervals (Fig. 7; Eq. 3). This amounts to a threefold increase of volumetric channel-forming discharge during HA intervals. The most conservative discharge estimates are obtained when using channel plug and lateral accretion width estimates such that HA intervals have discharge rates of 700 ± 150 [m^3^ s^−1^] (average value ± standard error, N = 45) while LA intervals have a total discharge of 200 ± 50 [m^3^ s^−1^] (N = 49).

Unit bedload sediment flux estimated using the Meyer-Peter and Muller equation, is estimated to be 1.7 ± 0.2 [kg m^−1^ s^−1^] (N = 45) and 2.0 ± 0.1 [kg m^−1^ s^−1^] (N = 49) for HA and LA intervals respectively (supplementary material Figure S6). Multiplying unit bedload sediment flux by channel-belt width (Eq. 4) results in total sediment flux of 300 ± 50 [kg s^−1^] for HA intervals (0.3 ± 0.05 [m^3^ s^−1^]), and 200 ± 20 [kg s^−1^] for LA intervals (0.2 ± 0.02 [m^3^ s^−1^]). This represents a 1.5-fold increase in bedload sediment flux during HA intervals (Fig. 7). Considering the additional preservation of floodplain material during LA intervals, these results suggest a significant increase of sediment transport during the highly amalgamated HA intervals, and also predict commensurate export of this clastic material downstream of the Escanilla fluvial system (e.g. to shallow-marine environments).

## Discussion

These results provide new insights into how palaeoslopes and palaeohydrology of the Middle Eocene Escanilla system evolved with alluvial channel stratal architecture. We find that the channel-belt palaeoslope is systematically lower in HA intervals and higher in LA intervals, and that this corresponds to greater (resp. lower) water discharge and bedload sediment flux during HA (resp. LA) intervals (Fig. 7). Moreover, unit discharge estimates, which are width independent (i.e., independent of the estimated channel-belt widths), also document a systematic increase of streamflow during HA intervals, and decrease during LA intervals (Figure S5). Currently, the hypothesis to explain the observed fluvial architecture in Olson is that it results from base level (accommodation variations) driven by local/regional tectonics in the Ainsa piggy-back basin^34^. However, such hypothesis (1) requires a, yet to be documented, physical mechanism that would account for tectonic oscillations with a periodicity of the order of 10^5^ years to explain the observed stratigraphic changes (Fig. 2c), and (2) does not account for the documented oscillations of water discharge. Similarly, accommodation variations could also be driven by (climate-controlled) eustatic sea-level changes. Although the magnitude of eustatic changes in the upper Eocene greenhouse is not well constrained, Huyghe et al. (2012)^49^ have suggested oscillations of the order of 20 m in coeval successions near the South-Pyrenean frontal thrust. However, (1) it is not clear how such eustatic changes would propagate upstream from the shoreline into the alluvial realm (e.g. Burns et al. 1997)^50^, (2) a eustatic control would not account for the observed channel discharge evolution, and (3) it would also imply an opposite relationship between aggradation and slope (Fig. 2a), i.e. alluvial slope would decrease with base level rise during LA intervals (and conversely, river bed slope would increase during periods of low accommodation). Instead, our observations taken together suggest that the stratigraphic architecture in this alluvial depositional zone is rather controlled by upstream factors which modulate water discharge variations, sediment transport, alluvial style and stacking pattern (Fig. 7). We discuss below the possible origin of such variations.

Upstream forcing of water and sediment fluxes can be associated to tectonic and/or climatic changes, which are the two allogenic controls primarily influencing depositional systems in the continental domain [e.g.,^51–53^]. Tectonic activity mainly influences sediment flux, normally without altering water discharge, except in case of structural disruption/modification of the drainage network in the catchment zone. Climatic changes, on the other hand, primarily affect precipitation, evaporation, overland and channel flow, which all influence the erosive and transport capacity within the drainage system, and thus determine the calibre and load of the fluvial sediment flux^53,54^. The effect of climatic changes on the production and release of sediment from source areas has been modelled in many studies [e.g.,^52,55–57^], and a link between climatic oscillations and fluvial architecture through variations in water discharge, sediment supply, and sediment size has also long been proposed in numerous works [e.g.,^58–61^. Thus, we postulate that a plausible explanation for the fining-upward trend of each HA-LA sequence could be a result of periodically decreasing precipitation (high during HA) and associated sediment discharge. With decreasing channel discharge, the fluvial equilibrium profile would be forced towards a greater gradient (as in Fig. 2b), driving fluvial aggradation, floodplain preservation and vertical stacking of channels (LA intervals). On the contrary, during periods of enhanced channel discharge, the river system would be forced toward a lower equilibrium bed slope (Fig. 2b), driving lateral mobility, amalgamation of channels and inhibiting vertical aggradation (HA intervals). At present, it remains unclear exactly what climatic conditions prevailed during the periods when we document larger (respectively lower) channel discharge. Indeed, the estimated larger discharges within HA intervals do not necessarily correspond to more humid conditions because drier climates with enhanced seasonality can also lead to channel-forming discharge of larger magnitudes. This has been suggested for similar situations during the PETM in Spain [e.g., Chen et al., 2018^62^] and in the Piceance basin, Colorado, USA, where Barefoot et al.^63^ documented increased channel mobility associated with enhanced seasonality. Further work, for instance using pedogenic and geochemical climatic proxies, will be needed to explore this issue.

The suggestion that climate has an important influence on stratigraphic architecture in the Escanilla system is consistent with the work of Armitage et al.^6^ on a numerical modelling study applied to the Escanilla sediment routing system. These authors predicted increased sediment flux due to increased precipitation in the catchment area during the Middle Eocene Climatic Optimum (MECO), approximately around 40 Ma near the C18r to C18n transition, which corresponds roughly to the “Olson sheet”, i.e., the HA interval of sequence 2. We indeed document an increase in water discharge and sediment flux at that time, which could also correlate well with the pulse of deltaic progradation recently documented by Peris Cabré et al.^64^ in coeval MECO successions of the adjacent Jaca basin to the West.

### What drove climate variations?

The repetitive pattern of the three fining-upward sequences studied here points at a recurrence in the controlling forcings at their origin. Moreover, other studies in the area have described several similar sequences above and below those studied in the present work^33–35^, suggest that this cyclic control may have persisted over an extended period of more than 2.8 Ma according to current stratigraphic constraints. Cyclical oscillations of the orbital configuration of the Earth-Sun system over so-called Milankovitch periodicities constitute a plausible mechanism to account for the observed cyclic changes in water discharge in the Escanilla fluvial succession. Milankovitch cyclicity is known to have played a major role in driving climate change during Earth’s history [e.g.,^65,66^], in the Cenozoic [e.g.,^67^], and has also been invoked in several studies in the Spanish Pyrenees as an important factor controlling depositional sequences [e.g.,^68^].

**Figure 8:**
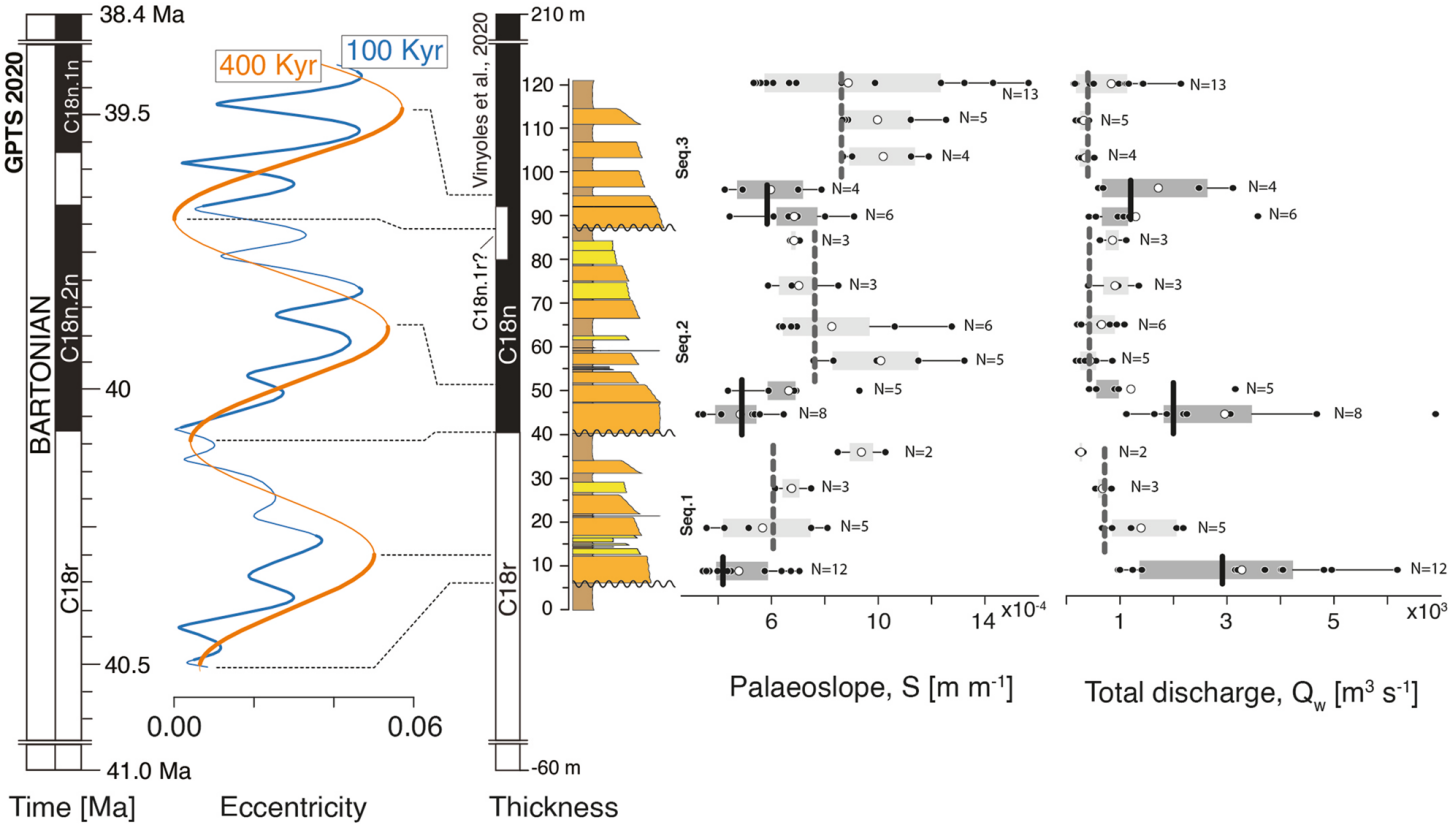
Eccentricity modulated water discharge variations in the Escanilla Formation. A plausible relationship between eccentricity cycles and water discharge variations which in turn control changes in stratigraphic architecture from HA to LA intervals such that palaeoslopes are lower in HA intervals and are higher in LA intervals.

To test this hypothesis, we tentatively calibrated the eccentricity model of Laskar et al.^69^ with the succession in Olson by matching the base (at 40 m on Fig. 8) and top (at 210 m) of Chron C18n (magnetostratigraphic data of^42^ to the base of Chron C18n.2n and top of Chron C18n.1n on the Geomagnetic Polarity Time Scale (GPTS 2020) (Fig. 8)^43^. The magnetozone C18n.1r has also been tentatively placed at 80–90 m, based on the presence of a thin reverse period yet with low/ intermediate quality paleomagnetic samples in the original dataset of Vinyoles et al.^42^ (Fig. 8). Although preliminary, this first-order scaling suggests a correlation between higher discharge HA intervals and phases of increasing eccentricity (400 ky signal, marked with a thick orange outline on Fig. 8), and conversely, lower discharge LA intervals correspond to phases of decreasing 400 ky eccentricity (Fig. 8). Such a correlation is consistent with the physical rationale according which periods of eccentricity maxima promote larger precession/insolation amplitudes more prone to trigger high-magnitude, extreme, events^70^, while decreasing eccentricity would instead diminish climate variability (seasonality)^71^ and the intensity of channel-forming discharge events. This scenario is also coherent with the study of Kodama et al.^72^ who demonstrated a systematic link between the 400 ky eccentricity maxima and increased terrigenous supply from fluvial source to coeval shallow-marine successions near Arguis in the neighbouring Jaca basin. Finally, such orbital control on stratigraphic cyclicity has also been shown to influence depositional style through control on siliciclastic sediment supply to the deep-marine Early-Middle Eocene Ainsa basin^73–76^ and may thus have been an important factor throughout an extended period of time in the South-Pyrenean foreland despite the important tectonic activity linked with mountain building at that time.

### Implications and conclusion

These findings have several fundamental implications for classical sequence stratigraphic predictions, sedimentary landscape evolution over geological timescales, the prediction of ancient hydroclimates during greenhouse forcing, and for industrial applications in resource exploration. First, our findings illustrate the dominant role of the sediment supply *S* on the stratigraphic architecture of a fluvial system, which contrasts with more classical hypotheses invoking a dominant role of accommodation *A* in determining fluvial stratal patterns [e.g.,^7,10^]. Second, our results suggest higher sediment fluxes during HA intervals, which is in agreement with classical sequence stratigraphic predictions in which such amalgamated packages are associated to low *A/S* conditions, and thus with greater down system transport of sediments to shallow-marine and deep-sea environments. Third, this work is also fundamental for our ability to reconstruct ancient hydroclimates from the sedimentary record and compare it to numerical model predicted response of the hydrologic cycle to warming conditions. For instance, modelling studies of the Palaeocene–Eocene Thermal Maximum (PETM) have suggested an intensification of the hydrological cycle on a global scale in response to greenhouse gas levels^77^. Results from this study are also consistent with the findings of Barefoot et al.^63^ where higher seasonality during the PETM increased lateral channel mobility. During the Middle Eocene greenhouse conditions of the Escanilla succession, HA intervals therefore record periods of increased lateral channel mobility linked with channel-forming discharge events (floods) of higher magnitude than LA intervals which present lower lateral channel mobility (lower magnitude channel-forming discharge events).

The framework of palaeoslope and palaeohydrological reconstruction across three fining-upward sequences in the Middle Eocene aged Escanilla Formation, for the first-time, documents lower river slope during higher water discharge and sedimentary flux with the deposition of river channels having a high degree of amalgamation, and higher slope during lower water discharge and sedimentary flux with the deposition of river channels having a low degree of amalgamation. The studied fining-upward sequences together with our finding of cyclic variations in water discharge and sediment flux represents a major paradigm shift by suggesting climate may have controlled the entire sedimentary landscape evolution during the Middle Eocene greenhouse conditions instead of eustatic variations, and with palaeohydraulic reconstructions to test different options without the need of an independent eustatic curve.

## Methods

### Data collection

Palaeohydrological field data were collected sequence-wise in a lateral spatial domain and from the respective HA and LA intervals. Data from channel fill deposits included channel basal gravels for grain size distribution and storey thicknesses as flow depth estimates. This data along with uncertainties associated with individual measurements were propagated through a quantitative framework to reconstruct hydrological parameters such as flow depths, palaeoslope, flow velocities, water discharge rates, bedload sediment flux. A Google Earth panel with the location of sampling stations has also been provided in the supplementary material.

Grain size measurements were collected using the Wolman sampling procedure^78^. The longest axis was measured as a proxy for the intermediate b axis on 100–200 grains per sampling station. The procedure was performed on photographs taken with a Canon EOS 2000D camera of 24.1 Mpixels resolution on an outcrop area of 1 × 1 m^2^. Grains were measured at the nodal intersection of a virtual grid such that a repeat count of grains is avoided. Measurements were made using ImageJ2 version 2.3.0/1.53f. The data obtained is normalized using the (psi) scale, a logarithmic scale with base two, to perform statistical analyses and obtain the 50th percentile (D_50_) of the grain size distribution.

Flow depth estimates are based on preserved storey thicknesses, channel-plug, and bar-scale clinoform heights measured using a laser range finder (TruPulse model 200) and following the procedure outlined in Mohrig et al.^79^ and Kelly^48^. It is important to note that while preserved thicknesses are lower than the original flow depths, preserved thicknesses do not severely underestimate the original depth^32,80^.

### Quantitative paleohydrology

Palaeoslopes were estimated using the empirical equation proposed by Trampush et al.^31^ (Eq. 1). We use this equation as our grain size measurements in a few instances are less than the 8 mm threshold required to use the Shields stress inversion approach^32^.

1 $$log \ S = {\upalpha }_{0} + {\upalpha }_{1} \, log\,D_{50} + {\upalpha }_{2} \, log\,H_{bf}$$

It is an empirical equation, motivated by theoretical considerations, and provides a relationship between the channel slope (S), median grain size (${\mathrm{D}}_{50}$), and bankfull depth (${\mathrm{H}}_{\mathrm{bf}}$). ${\mathrm{\alpha }}_{0}$, ${\mathrm{\alpha }}_{1}$ and ${\mathrm{\alpha }}_{2}$ are three empirical coefficients with values of − 2.08 ± 0.0015 (mean ± standard error), 0.2540 ± 0.0007 and − 1.0900 ± 0.0019, respectively. An average palaeoslope (± standard error) value has been estimated, per interval, using average median grain size values and average bankfull depths along with their respective standard errors. Average palaeoslope estimates are presented in [m/m], for example, a palaeoslope value of 0.001 represents aggradation of 1 m per 1000 m.

Channel-belt width W can be estimated using empirical scaling relations when direct measurements are not possible on the field. We estimate channel-belt widths using the relationship, W = 8.8H_bf_^1.82^^45^. Where possible, channel plug widths were estimated in the field using a laser range finder (TruPulse model 200) while widths from lateral accretion deposits was estimated using the procedure outlined in Greenberg et al.^44^.

Flow velocity, U was calculated using Manning’s equation (Eq. 2) where n = 0.03 ± 0.005 is the Manning’s coefficient, R is the hydraulic radius approximated by channel flow depths and S is slope. Flow velocity data can be found in the supplementary material.

2 $$U = \frac{1}{n}R^{2/3} S^{1/2}$$

Total water discharge ${Q}_{w}$ was calculated using Eq. (3)

3 $$Q_{w} = U \times H_{bf} \times W$$

For unit water discharge, W = 1.

Total bedload sediment flux Q_s_ was calculated using the Meyer-Peter and Muller equation (Eq. 4).

4 $$Q_{s} = \rho_{s} \left( {\Delta \rho gD_{50}^{3} } \right)^{1/2} C\left( {\tau^{*} - \tau_{c}^{*} } \right)^{3/2} \times W$$

where, ${sediment \ density \ \rho }_{s}=2650 \ kg/{m}^{3}$, buoyant density $\Delta \rho = 1.6$, constant $C=8$, critical sheer stress ${\tau }_{c}^{*}$ = 0.047 and shear stress ${\tau }^{*}=\frac{{H}_{bf}S}{\mathrm{\Delta \rho }{D}_{50}}$.

For unit bedload sediment flux, W = 1.

### Effectiveness of palaeohydrological reconstructions

While palaeohydrological information can be extracted for ancient systems, there remain concerns over its accuracy^29^. To address these concerns, we evaluate a few limitations in this study. Firstly, there is a possibility of under-estimating true depths based on storey thickness due to the incomplete preservation and erosion by the overlying storey. An under estimation of flow depth would consequently affect the slope, flow velocity, discharge, and flux estimates. Secondly, our total discharge and flux estimates are based on channel-belt widths. While individual channel widths can easily be determined for modern rivers, the inherent problem in doing the same for ancient systems, apart from low preservation, lies in determining the number of active channels. This could significantly affect our estimates such that a high number of active channels would imply significantly higher discharge and sediment transport rates.

### Statistical tests

Uncertainty on results reported in this study consist of the standard error of the mean (SE) calculated as $=\frac{SD}{\sqrt{n}}$ , where SD is the standard deviation and n is sample number. Uncertainty propagation was carried out using the uncertainties package on Python (Spyder version 4.0.1). Statistical analyses were performed on Python (Spyder version 4.0.1). To check for data normality, the Shapiro–Wilk test was performed using the *‘scipy.stats.shapiro’* package. The null hypothesis that the data is normally distributed cannot be rejected when the *p* value is greater than 0.05 at the 95% confidence level. To check for statistical significance, a two-sided *t* test was performed for normally distributed data using the *‘scipy.stats.ttest_ind’* package for the null hypothesis that two independent samples have an identical average value. For non-normally distributed data, a Kruskal–Wallis test was performed using the *‘scs.kruskal’* package for the null hypothesis that the median value of all groups is similar. The null hypothesis can be rejected when the p-value is less than 0.05 at the 95% confidence level. The degree of freedom (dof) was estimated as ‘(nx + ny) − 2’ where ‘nx’ and ‘ny’ are the lengths of the two independent parameters. Power analysis of *t* tests was performed, using *‘pingouin.power_ttest2n’*, to detect Type II errors. Pingouin is an open-source package written in Python 3^81^.

## Supplementary Information

Supplementary Information 1.

Supplementary Information 2.

Supplementary Information 3.

Supplementary Information 4.

## Data Availability

All data generated and analysed in this study, including locations of sampling stations have been provided as supplementary material files accompanying this manuscript.
