# Supplementary material for: Water discharge variations control fluvial stratigraphic architecture in the Middle Eocene Escanilla formation, Spain
Source: Sci Rep. 2023 Apr 26;13:6834. doi: 10.1038/s41598-023-33600-6 (PMC10133228; doi:10.1038/s41598-023-33600-6)
Supplement: Supplementary file 3 — Supplementary Information 3. [file 41598_2023_33600_MOESM3_ESM.docx]

**SUPPLEMENTARY MATERIAL**

**Water discharge variations control fluvial stratigraphic architecture in the Middle Eocene Escanilla Formation, Spain**

Nikhil Sharma^1,*^, Alexander C. Whittaker^2^, Stephen E. Watkins^1^, Luis Valero^1,3^, Jean Vérité^4^, Cai Puigdefabregas^5^, Thierry Adatte^6^, Miguel Garcés^5,7^, François Guillocheau^8^, Sébastien Castelltort^1^

^1^University of Geneva, Department of Earth Sciences, Rue des Maraichers 13, 1205 Geneva, Switzerland

^2^Department of Earth Science and Engineering, Imperial College London, South Kensington, London SW7 2AZ, England

^3^Paleomagnetic Laboratory CCiTUB-Geo3Bcn, Geosciences Barcelona–CSIC, C/Lluis Solé i Sabarís s/n, 08028 Barcelona, Spain

^4^LPG – Le Mans, UFR Sciences et Techniques, Université du Maine, 72089 Le Mans cedex 9, France

^5^Department of Earth and Ocean Dynamics, Faculty of Earth Sciences, Universitat de Barcelona, C/ Martí I Franquès, s/n, 08028 Barcelona, Spain

^6^Institute of Earth Sciences (ISTE), University of Lausanne, Bâtiment Géopolis, 1015 Lausanne, Switzerland

^7^UB-Geomodels Research Institute, Universitat de Barcelona, 08028 Barcelona, Spain

^8^Géosciences Rennes, Campus de Beaulieu, Université de Rennes 1, 35042 Rennes cedex, France

Correspondence to : Nikhil Sharma ([nikhil.sharma@unige.ch](mailto:nikhil.sharma@unige.ch))


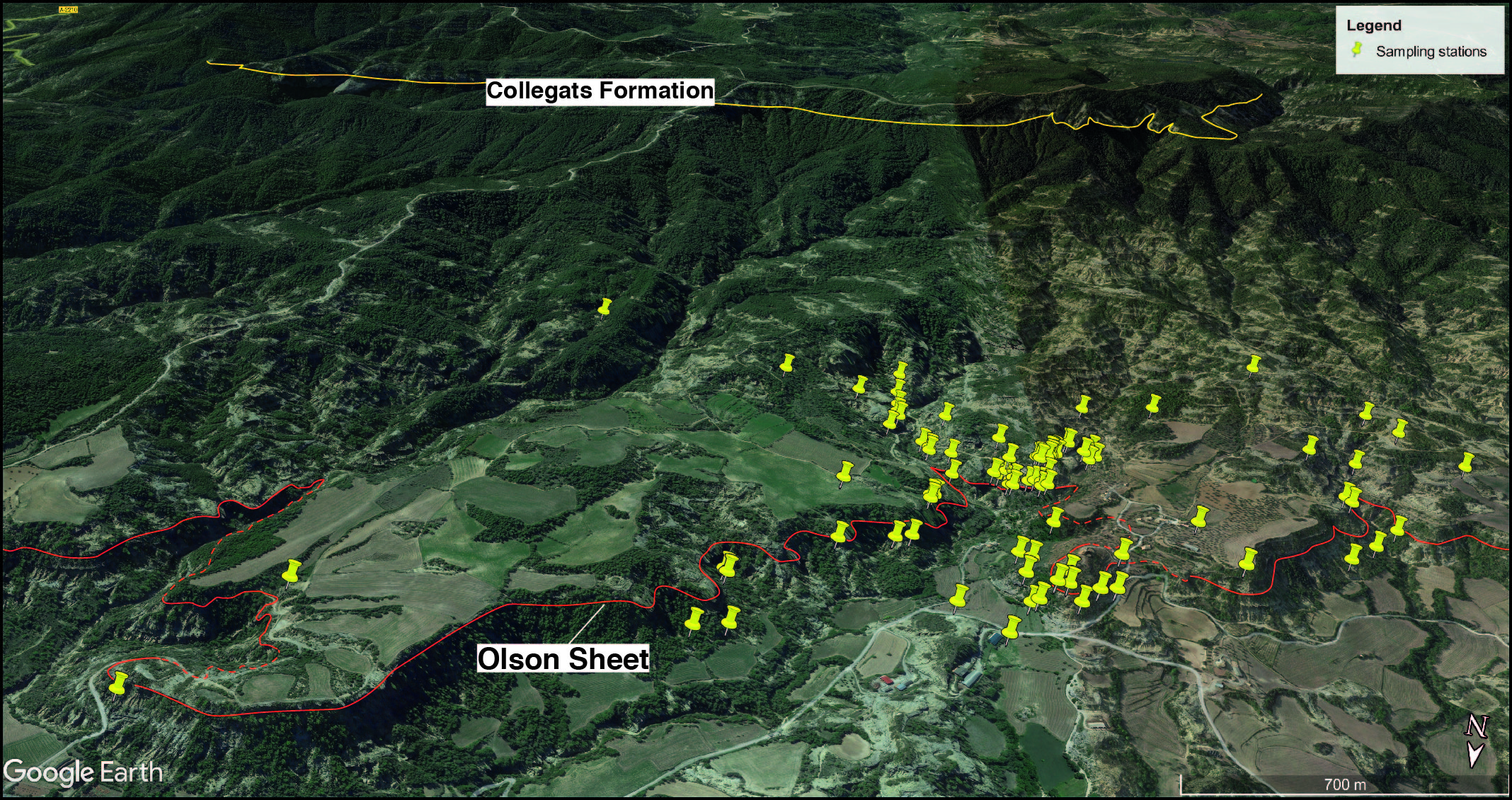


Fig. S1 **Google Earth panel with sampling stations.** A Google Earth panorama depicts the locations of sampling stations within the Escanilla Formation, Spain. Sampling stations are spread out in a lateral spatial domain for each HA and LA interval within a sequence. The ‘Olson Sheet’ and the Collegats Formation have also been marked on the panorama. The satellite map is from Google Earth Pro (https://www.google.com/earth/versions/).


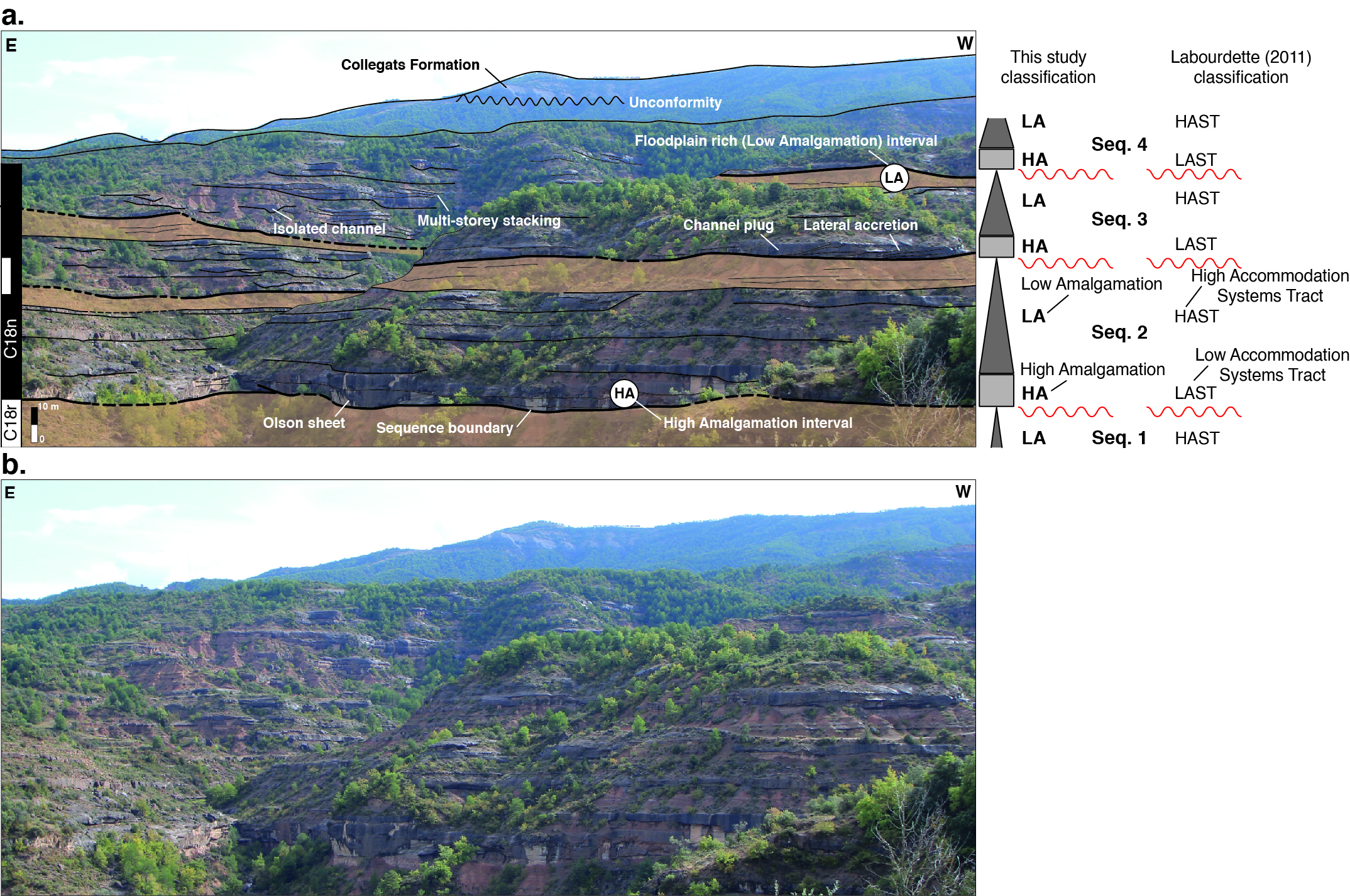


Fig. S2 **Panorama depicting the studied sequences containing High-Amalgamation (HA) and Low-Amalgamation (LA) intervals. a.** This panorama depicts the studied sequences 2, 3 and 4. Note that sequence 1 lies below the photographed interval and could not be captured. At the base of the panorama lies a thick floodplain rich interval above which lies a High Amalgamation (HA) interval containing the ‘Olson sheet’ and separated by a sequence boundary. Above the HA interval lies the floodplain dominated Low Amalgamation (LA) interval. Several stratigraphic features such as channel plug, lateral accretion, isolated channel, and multistorey stacking pattern have been marked as well. To the top of the panorama lies the Oligocene aged Collegats Formation separated from the underlying Escanilla Formation by an unconformity. An inset map has also been provided to make it easier for the reader to locate themselves along with a timescale marking the age of the photographed interval to the left of it. On the extreme right, the HA and LA classification used in this study is compared to the High Accommodation Systems Tract (HAST) and Low Accommodation Systems Tract (LAST) classification used by Labourdette (2011) **b.** An unannotated version of the panorama.


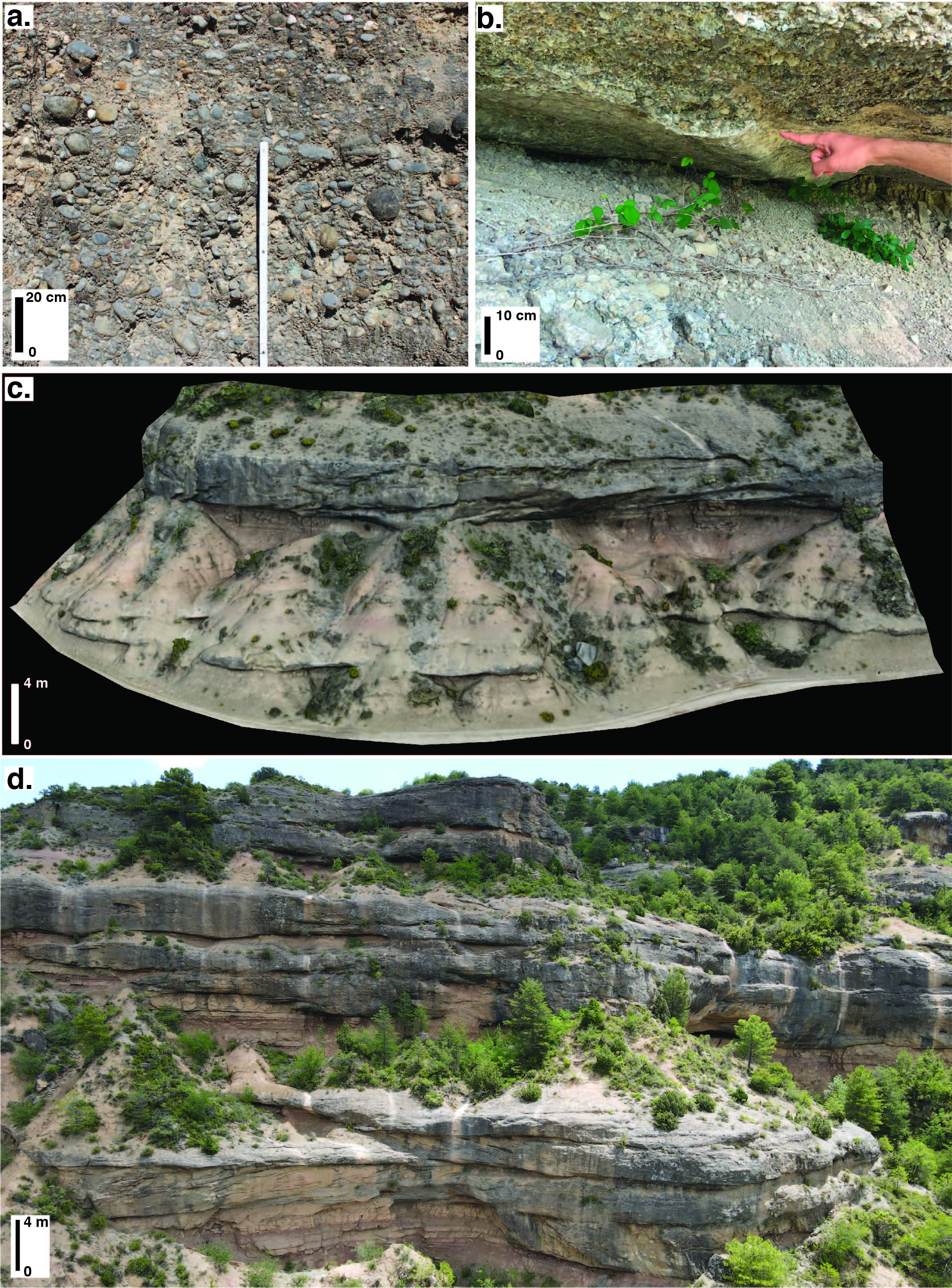


Fig. S3 **Unannotated version of outcrop photographs. a.** Channel basal gravel from which grain size estimates are obtained **b**. A large gutter used to reconstruct flow direction. It is marked as a long tube-like feature at the level of the erosive channel base lying over floodplain deposits **c.** A 3D model of an outcrop containing a channel plug and lateral accretion deposits, which illustrate the stratigraphic expression of H_bf_ **d.** Multi-storey stacking pattern observed in the Low Amalgamation (LA) interval of sequence 4. Several different stratigraphic features such as the different stories, accretion surfaces, storey bounding surface, bar bounding surface and floodplain relic are marked.


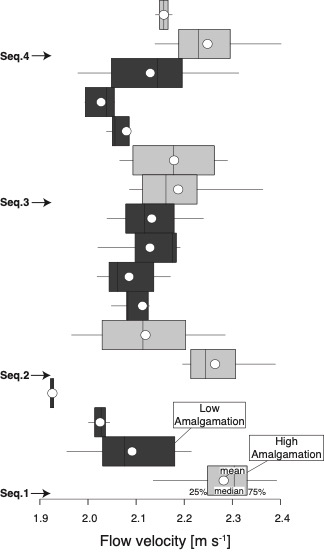


Fig. S4 **Flow velocity.** Flow velocity evolution across the sequences documented in this work. High Amalgamation (HA) intervals have higher flow velocities than in Low Amalgamation (LA) intervals.


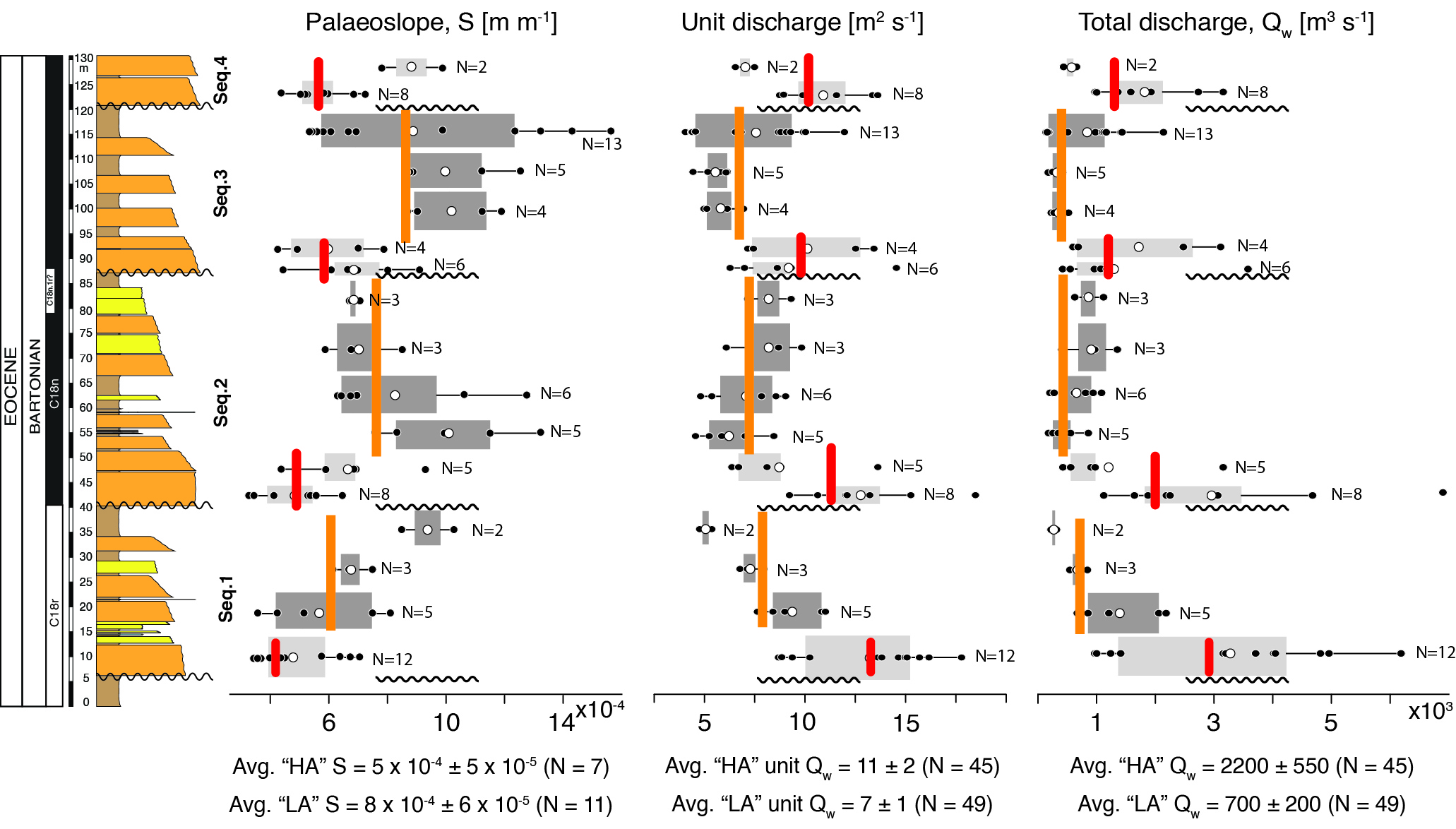


Fig. S5 **Palaeoslope, unit water discharge and total water discharge estimates.** Evolution in palaeoslope, unit discharge and total discharge estimates relative to each sampled storey within the HA and LA intervals of the studied sequences. Red bars denote the average value in the HA intervals while yellow bars denote the average value in the LA intervals. It is important to note the relationship and cyclical pattern, as shown by the overall average values in HA and LA intervals, between the three parameters such that river slope is lower when unit and total discharge are higher while river slopes are higher when unit and total discharge are lower.


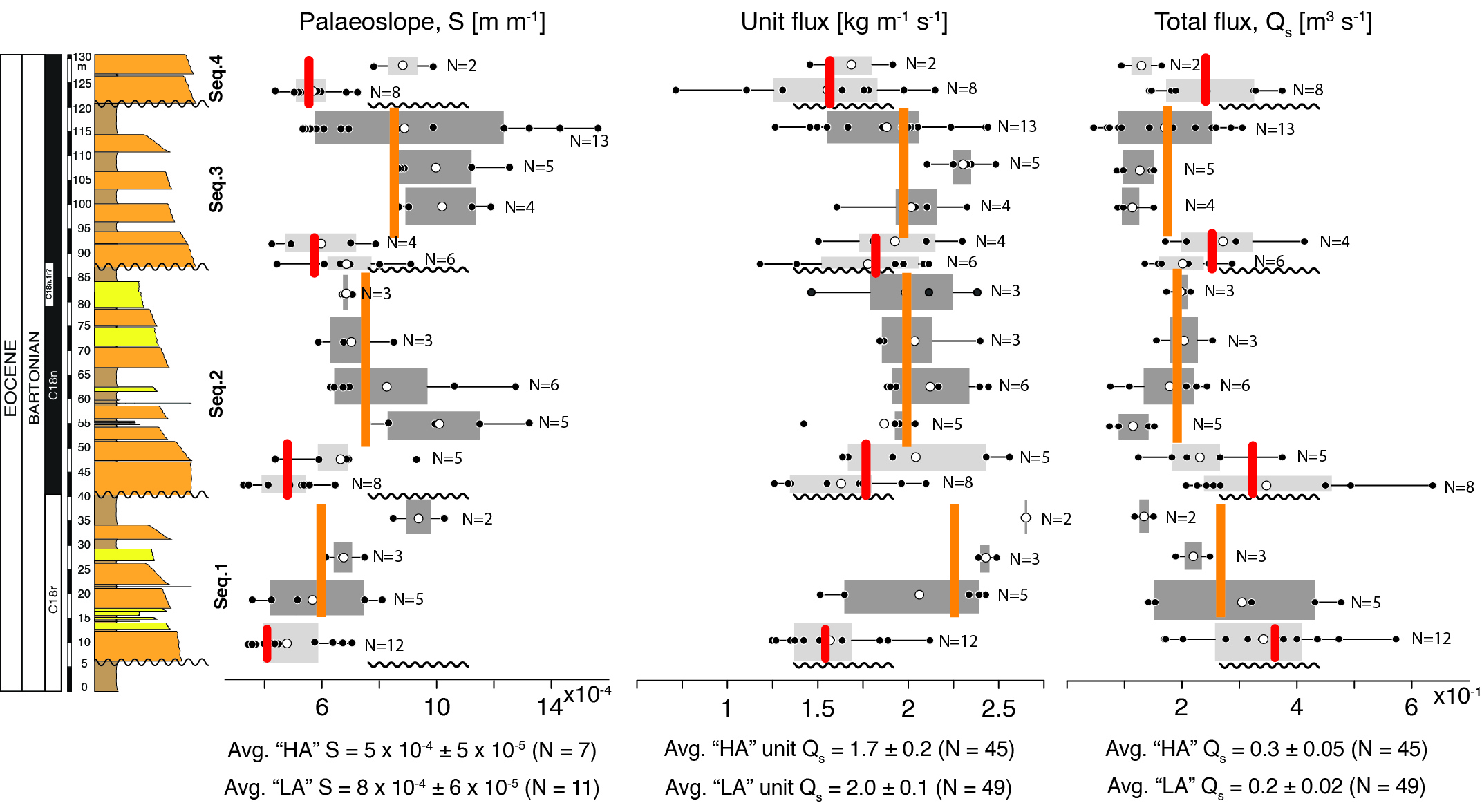


Fig. S6 **Palaeoslope, unit sediment flux and total sediment flux estimates.** Evolution in palaeoslope, unit flux and total flux estimates relative to each sampled storey within the HA and LA intervals of the studied sequences. Red bars denote the average value in the HA intervals while yellow bars denote the average value in the LA intervals.

Fig. S7 **Average total discharge estimates when using the different available width estimates.** Although absolute discharge estimates are different, the cyclical trend in water discharge variations does not change upon using different width estimates such that High Amalgamation (HA) intervals have higher water discharges when compared to Low Amalgamation (LA) intervals.

**References**

1. Labourdette, R. Stratigraphy and static connectivity of braided fluvial deposits of the lower Escanilla Formation, south central Pyrenees, Spain. *AAPG Bulletin* **95**, 585–617 (2011).
